# Ketamine plus Alcohol: What We Know and What We Can Expect about This

**DOI:** 10.3390/ijms23147800

**Published:** 2022-07-15

**Authors:** Natalia Harumi Correa Kobayashi, Sarah Viana Farias, Diandra Araújo Luz, Kissila Márvia Machado-Ferraro, Brenda Costa da Conceição, Cinthia Cristina Menezes da Silveira, Luanna Melo Pereira Fernandes, Sabrina de Carvalho Cartágenes, Vânia Maria Moraes Ferreira, Enéas Andrade Fontes-Júnior, Cristiane do Socorro Ferraz Maia

**Affiliations:** 1 Laboratory of Pharmacology of Inflammation and Behavior, Faculty of Pharmacy, Institute of Health Science, Federal University of Pará, Belém 66075110, PA, Brazil; nhkobayashi18@gmail.com (N.H.C.K.); sarah.farias@ics.ufpa.br (S.V.F.); diandra.arluz@gmail.com (D.A.L.); kissila.machado@ics.ufpa.br (K.M.M.-F.); breuscsta@gmail.com (B.C.d.C.); cinthia_bel@yahoo.com.br (C.C.M.d.S.); luannafe@hotmail.com (L.M.P.F.); sabrina_decarvalho@yahoo.com.br (S.d.C.C.); efontes@ufpa.br (E.A.F.-J.); 2 Laboratory of Psychobiology, Psychology Institute, University of Brasília, Campus Universitário Darcy Ribeiro—Asa Norte, Brasília 70910900, DF, Brazil; vmmf.unb@gmail.com

**Keywords:** ketamine, alcohol, drug abuse, addiction, toxicological effects, central nervous system, cardiorespiratory system, digestive system, renal system

## Abstract

Drug abuse has become a public health concern. The misuse of ketamine, a psychedelic substance, has increased worldwide. In addition, the co-abuse with alcohol is frequently identified among misusers. Considering that ketamine and alcohol share several pharmacological targets, we hypothesize that the consumption of both psychoactive substances may synergically intensify the toxicological consequences, both under the effect of drugs available in body systems and during withdrawal. The aim of this review is to examine the toxicological mechanisms related to ketamine plus ethanol co-abuse, as well the consequences on cardiorespiratory, digestive, urinary, and central nervous systems. Furthermore, we provide a comprehensive discussion about the probable sites of shared molecular mechanisms that may elicit additional hazardous effects. Finally, we highlight the gaps of knowledge in this area, which deserves further research.

## 1. Introduction

Drug abuse is an ancient human practice, which involves genetic and environmental factors, as well as a great complexity of neuronal circuitry [1]. Currently, the use of psychoactive substances has become a worldwide concern and public health issue, due to health risks and social problems [2,3]. In general, such involvement in drug misuse occurs early in life. On a global scale, it is estimated that 1 among 20 individuals, ranging from 15 to 64 years old, have already consumed any recreational substance to achieve altered states of consciousness [4,5].

Of note, ethanol consists of the most widely consumed psychoactive substance [6]. According to the World Health Organization (2014), the harmful use of ethanol is responsible for about 5.9% of deaths in the world, as a consequence of traffic accidents, social interaction problems, domestic violence, crimes, public disorder, and chronic health problems [7,8,9]. Moreover, the consumption of alcohol is a factor that can lead to the consumption of additional psychoactive drugs, such as methylenedioxymethamphetamine (MDMA), methamphetamine, lysergic acid diethylamide (LSD), gamma-hydroxybutyrate (GHB), and especially ketamine, which has been in evidence among drug abusers [10].

Ketamine has been used for non-therapeutic purposes, especially as a “club drug”, being referred to as “Key”, “Special K”, “angel dust”, “K”, or “Kit Kat” [11]. Ketamine is commonly misused by intranasal route, inhaled, or smoked [12]. When administered in liquid form, users inject it into the body or mix it in alcoholic drinks for oral route [13].

It is noteworthy that both psychoactive drugs share several mechanisms of action on the central nervous system (CNS). Primarily, ethanol evokes non-competitive inhibition of N-methyl-D-aspartate (NMDA) receptors [14,15,16]. Ketamine also acts through an NMDA open-channel blockade mechanism [17,18]. Although both drugs act equally on the same receptor, it is unclear whether ketamine can modify the molecular effects of alcohol on NMDA receptor [14,19]. Thus, we ask whether co-exposure to these substances could imply a synergistic toxicological effect mediated by the NMDA pathway, intensifying toxicological responses.

Several and robust preclinical and clinical studies have described the hazardous effects elicited by alcohol consumption, even after long-lasting withdrawal [20,21,22,23]. On the other side, ketamine non-medical purpose use has been described in only a few studies in the last decades [12,24,25,26]. Unfortunately, studies that have researched the consequences of ethanol plus ketamine concomitant exposure in body systems are scarce. Thus, we decided to collect available clinical and preclinical studies related to the recreational use of ketamine plus ethanol, focusing on its detrimental consequences. In addition, we propose the probable mechanism of the toxicological synergic effects that might contribute to hazardous effects of this co-consumption.

## 2. Epidemiological Features of Ethanol plus Ketamine Consumption

According to the 2018 Global Report on Alcohol and Health published by the World Health Organization (WHO), young adults, ranging from 20 to 39 years old, are the main consumers of alcohol [7]. In 2010, a survey reported that male alcohol intake was higher than that of females, with a consumption of around 19.4 L/year. In contrast, women consume about 7 L/year [27], but such a pattern of global alcohol consumption has been modified, since women have augmented alcohol drinking over the years in amounts that are increasingly closer to men [28]. Of particular interest is the co-ingestion of alcohol with other drugs by abusers [2,10,13].

Ketamine consists of a dissociative anesthetic applied to sedation and analgesia procedures and some psychiatry conditions [29,30,31]. Although there are few epidemiological data on the non-medical use of ketamine, it has been reported that ketamine misuse started in the 1970s [32]. It is used primarily in the United States, but nowadays the recreational use has spread worldwide [33]. Thus, ketamine is included as a new psychoactive substance (NPS) in drug-abuse criteria [11,31,34]. Currently, the Asian continent is emerging as a major geographic region that consumes this substance; ketamine is ranked as the first choice for misused drug among young abusers in Hong Kong. In Western countries, ketamine misuse is around 1–2% [35]. However, the recreational use of this drug has also increased in United Kingdom, Australia, and China [13]. In a French survey between 2012 and 2017, ketamine misuse was prevalent in 67% of NPSs detected [34]. From this information, we suggest that ketamine recreational use is not restricted to some countries; on the contrary, such psychoactive substance is overspread worldwide.

The concomitant use of ethanol plus ketamine has been described. In an emergency department of Bologna (Italy), alcohol was present with 25% of ketamine recreational misusers admitted to emergency care [36]. In accordance, the abuser’s co-ingestion percentage was around 39–98% in the preceding 12 months [37,38]. Unfortunately, both studies failed to link the negative symptoms to the possible synergism of toxicological effects of both drugs users were exposed to. However, a more prevalent association was found in controlled studies with volunteers recruited at party scenes, of which at least 65% of ketamine users also consume alcohol [24,39,40]. A wide survey reported that alcohol is present at a percentual of 98% of ketamine abusers, in a co-administration pattern or not [38]. Actually, all of these findings suggest the close relationship between ketamine and alcohol consumption.

### Fatal Outcomes

A positive relationship between recreational drugs plus alcohol and fatal or non-fatal overdose has been postulated [41,42,43].

In a wide Australian survey (2000–2019), ketamine self-administration as contributory to death was investigated [44]. In Darke and colleagues’ study [44], ethanol was present in a over a quarter (27.3%) of ketamine-related deaths. Previously, in the United Kingdom (1993–2006), alcohol was present in almost 50% of postmortem analyses from ketamine-related deaths [45]. Such few reports highlight the occurrence of polydrug use among addicts, in which alcohol consumption is prevalent. Of note, the limitation of the studies that clearly establish the contribution of each drug to a fatal outcome is obvious, since ketamine and alcohol are commonly associated with the use of other psychoactive drugs [44].

## 3. Body Systems Consequences of Ketamine plus Ethanol Abuse

### 3.1. Liver and Biliary System Damage

The recreational use of ketamine has been reported as a factor for eliciting abdominal pains related to unknown etiology (for a review, see Reference [46]). Such characteristic symptoms are colloquially referred to as “K-cramps” [47] and consist of several claims reported by ketamine abusers in emergency departments, with the occurrence of dilated common bile duct and choledochal cysts [48,49,50]. In addition, abnormal liver function and gallbladder dyskinesia have been found in clinical studies of ketamine abusers [48,49,51,52,53]. The exact mechanism that underlies ketamine hepatotoxicity is unknown; however, a clinical study has postulated that a direct toxic effect on parenchymal hepatic cells might occur [54]. Actually, an in vitro study revealed that ketamine induces hepatotoxicity through apoptotic mechanisms, specially reducing the mitochondrial membrane potential and ATP synthesis [55]. In addition, it is speculated that ketamine directly blocks the NMDA receptor and calcium channels’ smooth muscle cells of the biliary system; this may contribute to biliary dilatation [51]. The indirect mechanism of ketamine’s hepatobiliary hazardous effects relies on the inhibition of the dorsal motor nucleus of vagus through the NMDA blockade; this also leads to gallbladder dyskinesia [56]. All of the molecular hazardous effects mentioned above are related to ketamine’s pharmacological mechanism, which requires plasmatic concentration of this substance. Finally, ketamine’s pharmacokinetic profiles consist of a wide hepatic CYP P-450 inducer, which can trigger the toxicity of other drugs in the hepatobiliary system or other organs, through elevated production of toxic metabolites, which may last even during withdrawal [55].

On the other hand, the detrimental relationship between alcohol and the liver has been extensively reported (for a review, see Reference [57]). There are three main pathogenic mechanisms related to alcohol ingestion in the liver: oxidative damage, pro-inflammatory processes, and hepatocellular alterations elicited by ethanol and its metabolites [58]. In fact, there are direct hepatotoxic consequences of alcohol metabolites, mainly acetaldehyde, acetate, and fatty acid ethanol esters (FAEEs) [58]. Acetaldehyde and its subproduct, acetate, have a crucial role in alcohol-induced liver disease (ALD). Such metabolites are the products that result from the oxidation of alcohol by the cytoplasmic alcohol dehydrogenase (ALDH) enzyme, which, in general, is overexpressed in chronic ethanol consumption, followed by acetaldehyde dehydrogenase (ALDH), respectively, inducing toxicological liver effects and displaying hepatic fibrosis by a harmful feedback cycle, i.e., detrimental extra-cellular matrix remodeling, oxidative stress, and pro-inflammatory events [58,59]. All of these toxicological events occur as a result of blood alcohol concentration; however, they can persist during long-term withdrawal [23].

**Figure 1:**
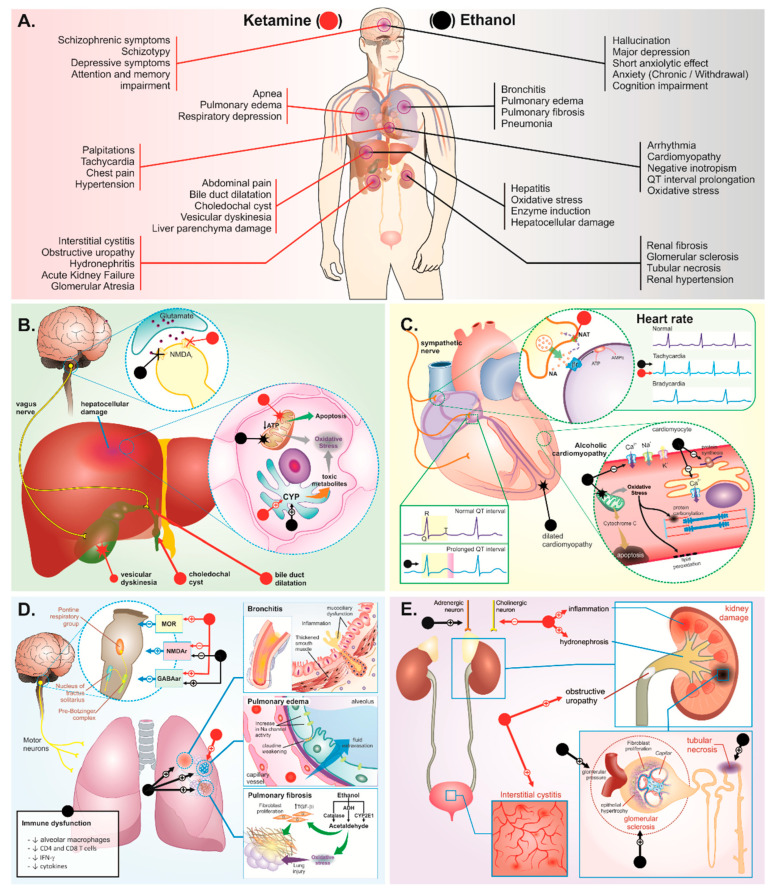
Schematic pharmacological targets with potential synergism between deleterious effects of ketamine (red markers) and ethanol (black markers), highlighting (**A**) the main clinical manifestations already described and effects related to (**B**) hepatic, (**C**) cardiac, (**D**) respiratory, and (**E**) urinary damage. (+) Activation, elevation, potentiation, or stimulation; (−) inhibition or reduction; (X) blocking; (✸) damage or disruption.

Although the epidemiological ketamine abuse surveys have not cleared the contribution of alcohol on gastrointestinal symptoms, such reports have highlighted that the co-abuse with alcohol has been prevalent (around 30%) [36,48,60]. Considering the findings above, we suggest that the co-abuse of ketamine plus ethanol by misusers may elicit cirrhosis, as well as increase collagen fibers in the liver [61,62] (Figure 1B). Actually, an experimental study revealed that high levels of cell death via a necrosis process have been detected in the livers of rats that received a ketamine-plus-ethanol regiment [61]. In addition, solely a case-report study has described the hepatobiliary symptoms observed in ethanol plus ketamine co-abuse [63], thus highlighting the non-relevance/non-importance of such co-abuse among clinicians.

### 3.2. Cardiorespiratory System

Epidemiological findings have reported the cardiovascular and respiratory systems’ consequences in the emergency services related to ketamine recreational use [48,60].

Palpitations, tachycardia, chest pain, and hypertension are symptoms claimed by ketamine abusers in the emergency department; these symptoms are related to cardiovascular toxicity [46,53,60,64], which may be generated through the hyperactivation of the reflex sympathetic [64,65]. Actually, ketamine presents pleiotropic effects, in which the inhibition of cholinergic transmission, as well as the blockade of neuronal and non-neuronal reuptake of norepinephrine, induces a persistent adrenergic systemic response [65]. Indeed, oxidative stress may play an important role in the cardiotoxicity induced by ketamine [66]. These effects are related to the pharmacological mechanisms of ketamine bioavailability, in which oxidative unbalance might persist.

On the other side, the involvement of alcohol on cardiorespiratory system is well established [67]. Acute or chronic ethanol consumption and its metabolites in systemic circulation (i.e., acetaldehyde) elicit arrhythmias and cardiomyopathy [67]. Negative inotropic effects, cardiac contractile dysfunction, myofibrillar structure alteration, and prolonged cardiac repolarization (QT interval) effects resulting from calcium/calmodulin-dependent protein kinase II (CaMKII) signaling hyperactivation, reactive oxygen species (ROS) overproduction, and cardiac ions homeostasis disturbance lead to cardiac failure [67].

Such cardiac toxicological overlapping may explain the increase of the toxicological evidence found in the co-ingested drugs [64]. Figure 1C shows the probable synergic toxicological mechanisms related to ketamine plus ethanol consumption on heart injury.

In the respiratory system, apnea, pulmonary edema, and respiratory depression have been observed in humans after ketamine misuse, as a result of its pleiotropic pharmacological actions [46,64]. In neurotransmitter systems, ketamine increases serotonin, norepinephrine, opioid, and GABAergic pathways’ activation [65], affecting ventilatory responses, which depend on the neurons or receptors activated (i.e., α2-adrenergic receptor), that can display depression of respiratory motor activity [68]. Ketamine is a weak agonist of the mu-opioid and GABAa receptors; however, at high doses, it increases GABA signaling [65]. It is well documented that mu-opioid and GABA receptors are involved in respiratory depression [46,69]. In addition, NMDA receptors plays a pivotal role in regard to ventilatory responses, in which the blockade of NMDA receptors also contributes to respiratory depression [70]. We suggest that synergic mechanisms of the multiple sites of ketamine action may induce respiratory system depression when higher plasmatic concentrations are reached [65].

In addition to the above effects, ethanol exposure also predisposes to lung injury (i.e., bronchitis, pulmonary edema, fibrosis, and pneumonia), primarily through oxidative damage [71]. Reduction of pulmonary innate defense mechanisms and adaptative immune responses have been displayed by mucociliary clearance dysfunction, impaired alveolar macrophages phagocytosis, decreased CD4+/CD8+-T cells and interferon-γ (IFN-γ) mediator, and breakdown of the protective cytokine signaling responses (for a review, see Reference [71]). Epithelial permeability of the respiratory airway is affected by ethanol, inducing pulmonary oedema through ion (Na^+^, Cl^−^, and K^+^) transport dysfunction, disturbance of claudin protein homeostasis, eliciting weakness of tight junction and pulmonary barrier [71]. Moreover, the increase of extracellular matrix deposition and overactivation of transforming growth factor beta 1 (TGF-β1) and metalloproteinases are responsible for fibrosis in the lung [71]. Such accessory detrimental effects emerge as a consequence of alcohol abuse in a long-term period in withdrawal.

As explained in regard to the cardiotoxicity features, we hypothesize that distinct, as well as similar, detrimental mechanisms in the respiratory system may occur, thus increasing the risk of respiratory depression from the co-ingestion of ketamine plus ethanol [44,64] (Figure 1D).

### 3.3. Urinary System

Urinary-system-related disorders have been correlated with the recreational use of ketamine [36,38,46,48]. The lower urinary tract has been the most affected region (early symptoms); interstitial cystitis presents the main evidence of ketamine misuse [48,53,72]. However, chronic ketamine use leads to more intense urinary symptoms and upper-tract involvement, such as obstructive uropathy and kidney damage [38,53]. Although both acute renal failure and hydronephrosis occur among ketamine abusers, such effects frequently are recovered during ketamine withdrawal [53,73]. The pathophysiology that underlies the ketamine-induced negative effects on the urinary tract is not well established; however, inflammatory abnormalities and the accumulation of precipitated ketamine metabolites in the pelvicalyceal systems may produce a direct toxicological effect on the urothelium, mainly at high plasmatic concentrations [53,74]. In a rat model, prolonged exposure to high dose of ketamine resulted in infiltrative inflammatory processes in the bladder and kidney, wherein the kidney seems to be more vulnerable at lower doses, and its abnormalities are more persistent [75]. In fact, controversial findings postulate that such an infiltrative process is induced by alterations in ion channel of the cells (i.e., Na+ channels, Ca^2+^-activated K^+^ channels, adenosine triphosphate-sensitive K^+^ channels, and Hyperpolarization-Activated Cyclic Nucleotide (HCN)-1 channels), but not by a ketamine deposit itself [76,77,78,79]. We hypothesize that an inflammatory process has been elicited by both mechanisms, the immune response and the disruption of ion channels’ homeostasis also resulted from the chemical presence of ketamine and its metabolites on the urinary system. In addition, in the long term, even in withdrawal after a long-lasting ketamine exposure, neurogenic damage associated with the loss of cholinergic neurons and neurotransmission reduction, as well as abnormalities on purinergic pathway, also underlie cystitis and bladder disruption [80,81].

Although there are conflicting theories related to the hazardous effects of ethanol on the urinary tract, the kidney seems to be the structure that is most vulnerable to alcohol’s detrimental effects. Regarding the lower urinary tract, robust evidence about the relationship between the deleterious effects of alcohol and ureters and the bladder is still scarce [82]. Thus, we focused on the upper urinary tract, specifically the kidney. In fact, epidemiological studies have linked heavy alcohol consumption as a modified risk factor for the development of renal damage [83,84]. Herein, experimental studies have investigated the pathophysiological features that underlie alcohol-induced kidney disorders [85]. Firstly, chronic alcohol exposure induces renal-structure changes, compatible with glomeruli atrophy, tubular necrosis, and alterations on renal tubules’ epithelia that can progress to renal fibrosis and glomerular sclerosis, primary induced by persistent hypertensive status and endothelial impairment [85,86,87,88]. In addition, renal function is mainly affected by hyperactivation of renin–angiotensin system, specifically mediated by the overexpression of angiotensin II and its receptor (angiotensin II type 1 receptor-AT1R), upregulation of the renal sympathetic pathway, and oxidative stress [85,89,90,91,92]. Besides the loss of renal homeostasis dysfunction, systemic and local elevation of blood pressure and renal failure occur in the long term, even in withdrawal [85,89,90,91,92].

Although ketamine abuse has been accompanied by alcohol intake among misusers, few clinical studies have underlined the toxicological effects that result from the association, focusing mainly on the urinary system [37,38]. In an experimental study, ketamine-plus-alcohol-treated animals developed atresia of glomeruli and necrotic cell in the kidney related to proteinuria, which infers renal dysfunction [61,62]. Such evidence suggests that ketamine–alcohol co-treatment augments the toxicological effects of both drugs per se, reducing the period of exposure to elicit nephrological impairment [62]. However, it is unclear the pathophysiology of such toxicological synergic mechanisms. We hypothesize that pro-inflammatory-pathway triggering of oxidative stress, ion channels’ homeostasis disruption, and elevation of renal blood pressure may underlie kidney damage in ketamine plus ethanol abuse (Figure 1E).

Finally, despite that the main molecular mechanisms investigated upon ketamine and alcohol occur in the CNS, the findings above highlight that these substances may affect other important organic systems; furthermore, they reinforce that peripherical alteration can elicit behavioral alterations. Both clinical and pre-clinical studies have shown that ketamine can display peripheral unbalance; however, clinical studies have key features that need to be pointed out. Works have failed to explore ketamine per se among users, since polydrug use is common in addiction. Moreover, it is evident that such a gap in the distinction of misused substances does not permit us to establish adequate drug-effect identification. Thus, experimental studies claim to identify such detrimental effects and have reported the occurrence of renal, hepatobiliary, cardiovascular, and respiratory damage, in addition to CNS injury discussed later. Clinical studies on ethanol’s harmful effects have been well documented, considering that alcohol consumption per se frequently occurs.

To facilitate the visualization of these changes, as well as to compilate relevant information about such relevant studies discussed until this point in the paper, we summarize, in Table 1, the effects of ketamine and/or ethanol on the liver, biliary, cardiovascular, respiratory, and urinary systems.

### 3.4. Central Nervous System

Ketamine CNS effects have been extensively described, primary by its psychotropic therapeutic use. Among recreational users, such a psychoactive drug at subanesthetic doses induces dissociative effects (also called “K-hole”), hallucinations, and altered status of consciousness, which favors the searching for this substance [93,94]. CNS consequences displayed by ketamine’s abuse range from cognitive-function disabilities to psychiatric disorders [93].

On the other side, as extensively discussed elsewhere, alcohol abusers undergo several behavioral alterations, thus reflecting its widespread toxicological effects on CNS, with long-lasting negative repercussions [95,96].

#### 3.4.1. Behavioral Disorders

##### Psychosis

Curran and Morgan’s research group has extensively studied the hazardous effects of ketamine recreational use on the CNS [40,93,97,98,99,100,101,102,103,104,105,106,107]. Schizophrenic symptoms have been frequently reported in frequent users of ketamine [24,40]. The immediate effects after 30 min of ketamine consumption elicit markable levels of schizophrenia-like behavior in polydrug abusers, with magical ideation, perceptual distortion, and thought-disorder symptoms [24]. Of interest, in early withdrawal (i.e., 24 h or 3 days of abstinence), schizotypal symptomatology still persists, characterizing a residual repercussion [24,39,103]. These psychopathological features have appeared in frequent, as well as in infrequent, users, correlated to extensive use of ketamine [39]. Such a ketamine-induced schizophrenic profile has been related to the reduction of NMDA receptors, and this elicits the NMDA-dependent negative symptoms observed among schizophrenic individuals who are free of clinical treatment [108,109,110].

The exact mechanism that involves schizotypy symptoms has been investigated. NMDA receptors’ blockade on parvalbumin-expressing (PV) interneurons results in disinhibition of pyramidal cells and increment of γ-band oscillations, reflecting cortical hyperactivity that plays a pivotal role in schizophrenia manifestation, related to positive symptoms such as hallucinations, delusions, disorganized speech, and catatonic behavior observed during ketamine bioavailability [111,112,113]. In this context, ketamine administration may display hyperactivation of glutamatergic circuitry in the brain that is associated with CNS overstimulation, resulting in behavioral and cognitive impairments, which reflect schizophrenic features [114,115,116,117]. In the long term, mechanisms of prevention of glutamatergic excessive transmission and excitotoxicity are dull, since the excitatory amino acid transporters 1 and 2 (EAAT1/2) expressed in astrocytes, which are responsible for the reuptake of glutamate, undergo downregulation after NMDA blockers’ exposure, which supports the hyperlocomotion and other positive characteristics in withdrawal related to schizophrenic patients [118].

Although the hyperactivation of glutamatergic circuitry plays of a vital role in ketamine-induced psychomimetic manifestations, dopamine receptor D2-like hyperfunction strongly contributes to schizophrenic symptoms [119]. In fact, animal model studies reveal that NMDA-blockers elicit positive schizotypy by enhancing neurotransmitters’ release, not only glutamate and dopamine, but also serotonin and acetylcholine, in the prefrontal cortex [108,120,121,122]. In addition to having an NMDA-receptor-dependent mechanism, ketamine also directly activates dopamine D2-receptor, with equal affinity observed in the NMDA receptor, which strongly contributes to psychotomimetic effects [123]. Moreover, studies have evidenced ketamine’s weak binding affinity for 5-hydroxytryptamine (HT)2A receptors. In fact, 5-HT2A activation by ketamine occurs through the direct interaction with this receptor, as well as the inhibition of voltage-gated K^+^ channel (Kv) currents’ conductance modulation, potentiating Kv inhibition mediated by 5-HT2A receptor, facilitating both membrane depolarization and 5-HT2A receptor signaling [29,124]. Furthermore, the acetylcholine transmission system increases and triggers the release of additional serotonin in the prefrontal cortex, and this may result in schizophrenic features related to serotoninergic hyperactivation pathway, resulting in visual hallucinations [122,125].

**Figure 2:**
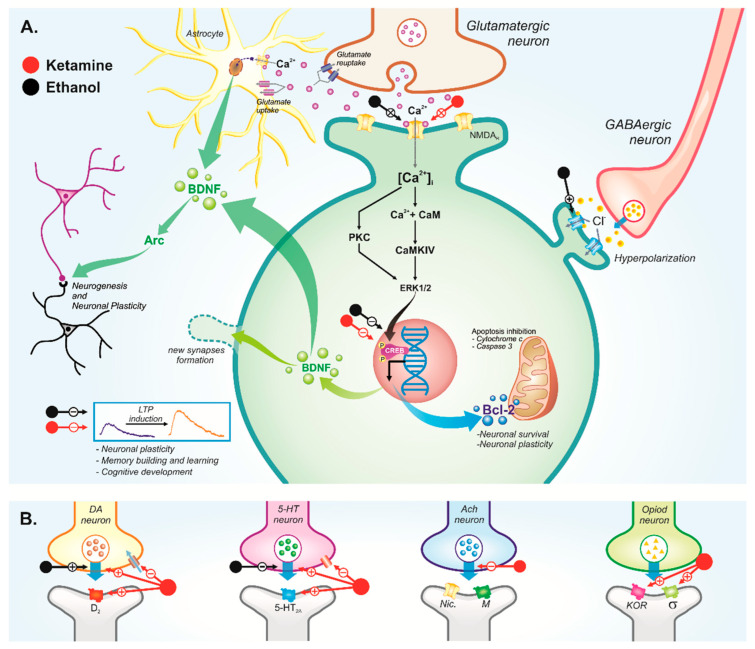
Principal pharmacological mechanisms and repercussions of ketamine (red markers) or ethanol (black markers) use during drugs bioavailability on (**A**) glutamatergic, GABAergic, (**B**) dopaminergic, serotoninergic, cholinergic, and opioidergic neurotransmission on the central nervous system. (+) Activation, elevation, potentiation, or stimulation; (−) inhibition or reduction; (X) blocking.

Opioid receptors represent other weak pharmacological targets implicated in the pathophysiology of schizophrenia [126]. The hypothesis that the sigma-receptor is involved in the schizotypy displayed by ketamine use relies on controversial studies demonstrating that postmortem schizophrenic-brained individuals present reduced levels of sigma receptors in the temporal cortex, but not in the parietal area [127,128]. The hyperactivation of sigma receptors (specially sigma-1 receptor) by phencyclidine (PCP)-like drugs may contribute to schizophrenic-like symptoms observed in ketamine abusers [126]. In addition, the kappa opioid receptor that displays psychomimetic properties presents ketamine binding affinity, which also may contribute to schizophrenic symptoms [129,130]. Finally, we hypothesize that promiscuous ketamine targets, primarily at NMDA, D2-type, and 5-HT2A receptors, as well as other weaker sites, may synergically potentiate psychotic effects in ketamine abusers (Figure 2).

Although alcohol psychotic-like symptoms are difficult to diagnose because of confounding elements (i.e., alcohol withdrawal delirium, which presents a short course of illness), it is well documented that alcohol addiction is an important aetiologic factor for psychosis, especially the auditory hallucinatory symptoms associated with delusions, as described since 1847 [131,132]. This condition is called Alcohol-Induced Psychotic Disorder by the American Psychiatric Association (DSM V-TR, 2000) [133]; the discussion relied on whether alcohol acts as a trigger factor for schizophrenia in a latent form [134]. This axiom has been rejected after differential diagnosis proposed by specialist authors [135]. Anxiety, auditory hallucinations, and delusions of persecution are the features related to alcohol hallucinosis syndrome, which can result in paranoid psychosis [132]. The pathophysiology of Alcohol-Induced Psychotic Disorder presents several gaps. Few image studies show the frontal, thalamic, basal ganglia, and cerebellar regions’ reduced activity during psychotic symptoms in withdrawal [136,137].

Alcohol-induced hallucinations share several of the neurobiological events elicited by psychotic-like ketamine-induced symptoms. During both ethanol and ketamine blood concentrations, dopaminergic circuitry hyperactivation, serotonin brain reduction, and reduced inhibitory transmitters (i.e., GABA and glycine) contribute to hallucinations displayed by chronic alcohol intake [138,139,140] (Figure 2).

**Figure 3:**
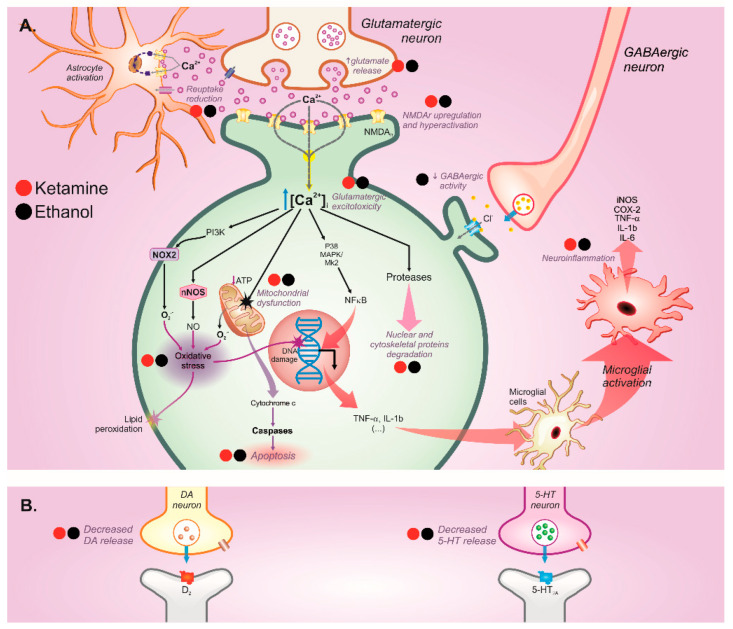
Principal repercussions of ketamine (red markers) or ethanol (black markers) withdrawal on (**A**) glutamatergic, GABAergic, (**B**) dopaminergic, and serotoninergic neurotransmission on the central nervous system. (✸) Damage or disruption.

Furthermore, ethanol and ketamine withdrawal display hyperactivation of the brain excitatory pathway after a prolonged blockade of excitatory receptors (especially NMDA receptors) [138] (Figure 3). We suggest that these overlapping molecular toxicological mechanisms may synergically contribute to psychotic-like effects among users of ketamine in associated with alcohol. Actually, a preclinical study demonstrated that co-exposure of ketamine plus alcohol for 14 consecutive days increased the release of dopamine and glutamate in the cortex and hippocampus in relation to hyperlocomotion, which characterizes the schizotypy profile during both drugs’ bioavailability [141]. Moreover, alcohol-potentiated neurotoxicity is induced by ketamine administration through mitochondrial dysfunction; brain-derived neurotrophic factor (BDNF) signaling impairment; and inhibition of cyclic AMP-responsive element binding protein (CREB) pathways’ signaling factors, i.e., serine/threonine kinase (Akt), calmodulin-dependent kinase IV (CaMKIV), and protein kinase A (PKA), along with reflexes on apoptosis, synaptic plasticity, and neuronal growth [141] (Figure 3). Of note, the influence of co-exposure exhibits distinct responses related to dopamine release, as well as BDNF expression, according to the specific brain tissue and the dose of both toxicants [142].

##### Depression

Although ketamine’s antidepressant effects have been explored, a longitudinal study among non-medical users has reported that mood depression disorder was prevalent even after a prolonged absence of the drug [40]. In fact, in a relevant controlled clinical study conducted among ketamine non-medical users, Morgan and colleagues found that frequent users (i.e., ketamine self-administration at least four times/week) presented depressive symptoms in withdrawal, at a mild-to-moderate scale, in a dose-dependent manner [39]. In a subsequent study in withdrawal, the authors showed that depression disorder was present in both frequent and abstinent ketamine users, reflecting changes in longitudinal emotional effects, even in the absence of depressive features previously detected [39,40]. Such relevant findings highlight the risk of the development of additional long-lasting psychological disorders across the time after ketamine misuse, correlated to a pattern of self-administration, which is, curiously, since ketamine has been claimed to have prolonged antidepressant activity in depressive patients [105]. It is noteworthy to mention that, even at therapeutical doses, ketamine elicits psychotic symptoms in healthy volunteers, thus suggesting its potential to modulate mental status in a way that leads to disordered thinking and behavior in healthy individuals [108]. Nonetheless, under abuse conditions, the frequency of consumption, dose, route of administration, and co-administration with others psychoactive substances are relevant factors that affect the negative impairments observed [108].

The pathophysiological mechanism that underlies depression symptomatology linked to the recreational use of ketamine appears to be a result of combinate mechanisms. Since ketamine acts as an antagonist of NMDA receptors, a compensatory increase of glutamate activity may occur, as usually observed in NMDA-blocker drugs, including ethanol, resulting in excitotoxicity in withdrawal period [143,144,145] (Figure 3). In turn, excitotoxicity underlies neurodegenerative processes, marked by microglial activation and astrocytic hypertrophy, which culminate in increased levels of pro-inflammatory cytokines, induction of cyclooxygenase 2, upregulation of neuronal nitric oxide synthase, and oxidative stress in animal models [146]. All of these processes have been claimed as molecular mechanisms that elicit depression [147,148,149,150] (Figure 3). Actually, our group showed that ketamine withdrawal elicits depressive-like behavior associated to hippocampal oxidative damage, thus suggesting that oxidative stress plays a role in the neurobehavioral impairment induced by the ketamine subanesthetic paradigm in animal models [25].

On the other hand, ketamine also induces the activation of α-amino-3-hydroxy-5-methyl-4-isoxazolepropionic acid (AMPA) receptors, triggering the expression of their subunit GluA1, as well as stimulating the mammalian target of rapamycin (mTOR), which consists of mechanisms involved in synaptic plasticity and synaptogenesis [108]. Bioavailable ketamine inhibits NMDA receptors and upregulates AMPA receptors on medial prefrontal cortex pyramidal cells, resulting in the activation of projections to dorsal raphe nucleus and serotonin release [108] (Figure 2). Thus, it is reasonable to infer that ketamine bioavailability and withdrawal affect neuronal plasticity and decrease serotonin signaling, which directly affects emotional homeostasis (Figure 2 and Figure 3).

The literature has proposed that ketamine inhibits the neuronal reuptake of norepinephrine, dopamine, and serotonin [65]. The increased levels of dopamine and noradrenaline observed after ketamine administration suggest the existence of stimulatory projections to the ventral tegmental area and locus coeruleus [108], as illustrated in Figure 2. Particularly, an increase of dopamine activity in the *Nucleus accumbens* is involved in addiction mechanisms of several drugs’ abuse, according to the cerebral reward system theory, which proposes that the absence of these psychoactive substances consequently induces depressive symptoms [151,152] (Figure 3). In fact, ketamine per se increases dopamine levels related to rewarding behavior in a place-preference task, which characterizes the potential dependence effect of this drug [19] (Figure 2).

In addition, depression has been extensively documented during withdrawal among alcohol abusers in clinical studies, as well as in animal models. It has been proposed that the overstimulation of NMDA receptors occurs in major depression [108]. Moreover, studies report compensatory effects of glutamate activity during ethanol abstinence, which induces neuronal excitotoxicity through NMDA receptor hyperactivation [95,143,144,145,153,154,155] (Figure 3). As mentioned before, excitotoxicity results in neuronal degeneration and neuroinflammation, in which such disturbances have been widely reported in depression pathology [108,146,148,149]. Indeed, depression symptomatology has been associated with cytokine and chemokine expression levels’ upregulation in the saliva and plasma of individuals that consumed ethanol, as well as dysbiosis of salivary microbiota [156]. Furthermore, a degenerative process provoked by excitotoxicity is accompanied by mitochondrial damage, release of superoxide radicals, lipid peroxidation, and other mechanisms linked to oxidative stress [147,150]. In experimental studies, our group found depressive-like behavior during withdrawal in rats submitted to binge-drinking protocol during adolescence, related to oxidative stress [21,157] (Figure 3). Robust evidence has proposed that depression pathophysiology is associated with reduction of neuronal viability, neuroinflammation, and oxidative stress, and may explain the occurrence of depressive profile among chronic ethanol users [95,146,147,148,149,150]. Finally, ethanol also alters the activity of several monoamines related to mood (monoamine theory), thus contributing to depressive outcomes [158,159,160,161] (Figure 3).

The isolated effects of ethanol and ketamine have direct or indirect potential to trigger depressive symptoms. However, few studies have explored the concomitant consumption consequences of these toxicants. Apparently, under the bioavailability of both of these drugs, reinforcement of compensatory effects in glutamatergic activity occurs, resulting in neuronal apoptosis stimuli and downregulation of important pathways of neuronal plasticity on cortex and hippocampus in animals [141]. As commented previously, hyperactivation of NMDA–glutamatergic signaling provokes excitotoxicity, which leads to apoptosis, neurodegeneration, neuroinflammation, and oxidative stress, including in brain areas related to emotional behavior, favoring depressive status in withdrawal [162,163] (Figure 3). In addition, disturbances in monoamines’ activities and synaptic plasticity may alter neurotransmission homeostasis, thus eliciting depression symptoms. In a 30-days ketamine-plus-ethanol challenge withdrawal, animals exhibited rewarding effects in a drug-abuse paradigm. [19]. This altered behavior was associated with dopamine levels being increased in then striatum, the upregulation of four dopamine metabolism genes mRNA levels, and BDNF overexpression on the cortex–striatum circuitry [19]. This augment of dopamine levels elicited by ketamine-plus-ethanol exposure also was reported in the hippocampus [141]. These findings are aligned with the monoamine theory of depression [164], and they also support the predictive potential rewarding behavior of these psychoactive drugs in co-misuse, overstimulating the rewarding pathway, and consequently developing depressive symptoms in long-lasting withdrawal (Figure 3).

In addition, chronic ketamine-plus-ethanol co-administration for 21 days in adolescent rats resulted in depressive-like behavior, an increase of apoptotic cells, and the upregulation of the expression of pro-apoptotic proteins caspase-3 and Bax in prefrontal cortex during withdrawal [164]. In Li et al.’s study, these negative effects were intensified by co-intoxication [164]. These data reflect the pro-apoptotic process as another neurotoxicological mechanism elicited by co-abuse in depression pathophysiology (Figure 3).

##### Anxiety

Anxiety is a complex emotional disturbance, and its pathophysiology involves central and autonomic responses; it is a common manifestation in CNS disorders. Among ketamine users, anxiety, accompanied by shaking and sweating, is a symptom that is manifested during withdrawal [93]. In a clinical study with ketamine users during a hospital detoxification treatment, ketamine chronic users exhibited moderate anxiety [165]. In an additional longitudinal study, ketamine users’ individuals presented high scores of anxiety profile in anxiety-test measurement [166]. In animal models, controversial data were found. Acute administration in the first hours of consumption elicits a rapid, but not sustained, anxiolytic response, while long-lasting evaluations and chronic use induced anxiety in withdrawal [167].

Our group showed that ketamine three-consecutive-administration protocol withdrawal elicits an anxiety-like profile in adolescent rats that is associated with oxidative damage in the hippocampus [25]. Oxidative stress and neuroinflammation are common components of the pathology of several CNS disorders. Considering the possible neuroadaptations due to the multiple systems affected by ketamine, especially the NMDA receptor blockade, excitotoxicity might occur (Figure 2). Damage in essential brain areas related to fear and alertness control (i.e., prefrontal cortex) under both drugs’ effects or withdrawal in animals was observed, for which the induction of apoptosis and reduction of neuronal viability may disrupt physiological mechanisms of anxiety inhibition [141,168] (Figure 2 and Figure 3).

Glutamatergic hyperfunction have been proposed to evoke anxiety-related symptoms in ethanol users during an abstinence period [143,154] (Figure 3). In addition, sympathetic and cortisol overactivity represent the consequences postulated by hypothalamus–pituitary–adrenal (HPA) axis activation theory, as elements of the pathophysiology of stress and anxiety [169]. In the ketamine literature, we did not find studies that investigated such pathway; however, some studies have demonstrated that the HPA axis is affected by ethanol exposure [96,170,171,172]. Finally, dysregulations in monoamine activities also can be speculated, since drugs that equilibrate the release and functions of these neurotransmitters are able to control symptoms manifested by these anxious patients [65,108,161]. Previously, we discussed the effects of both drugs on monoamines homeostasis, especially during withdrawal; however, it is necessary clarify if and how this imbalance occurs and contributes to anxiety manifestations. For example, ketamine induces an increase in monoamine release, and the opposite effect was expected in withdrawal [65] (Figure 2 and Figure 3). On the other hand, it has been proposed that serotonin transmission increases after acute alcohol administration and decreases during alcohol abstinence, and this might contribute to anxiogenic symptoms in withdrawal [161] (Figure 3).

Although studies have failed to prove the anxiogenic effect under ketamine plus ethanol bioavailability (1 h post-administration) or during withdrawal (24 h post-administration) [168,173], peculiar factors such as the dose utilized, treatment duration, and time of evaluation after intoxication may influence the results obtained; thus, additional studies are necessary to clarify the comprehension of potential toxicological effects of this co-abuse in relation to anxiety. We speculate that the convergence of toxicological mechanisms shared by ketamine and ethanol on anxiety neurobiology presents potential synergistic effects, which theoretically can intensify the pathophysiological mechanisms of anxiety, resulting in an amplification of anxiety symptoms. For example, synergic activities elicited by ketamine and ethanol resulted in neurotoxicity induced by glutamatergic pathway upregulation, as well as neuronal loss on cortical areas [83,141,144,145]. In fact, it remains unclear how these synergistic effects on the monoaminergic system contribute to anxiety.

##### Cognitive Disorders

Scarce studies have focused on ketamine and its consequences on cognition. Experimental studies have found that a single neonatal exposure to the therapeutic use of ketamine leads to short-term reduction in hippocampal cellular viability, as well as long-term alterations in hippocampal glutamate transport and short-term recognition memory prejudice in withdrawal [174]. Clinically, ketamine’s therapeutic use as an anesthetic agent in a pediatric patient in a pediatric intensive care unit displayed long-lasting disabilities in cognition [175]. In this sense, early exposure, even under therapeutic use, might provoke disruptions on cognitive domains, especially related to glutamatergic pathway inhibition.

In regard to non-therapeutic use, Morgan and colleagues have reported semantic and episodic memory compromising among ketamine misusers’ withdrawal, evaluated 3 days following drug use and in a follow-up after 3 years [105]. These authors observed that, even after the reduction of ketamine consumption over 3 years following the first assessment, impairments in attention and episodic memory still persist [105].

As mentioned previously, ketamine blocks NMDA receptors, which are responsible for crucial steps of memory formation, such as the long-term potentiation (LTP) process. LTP is classically described as being related to hippocampal neurons of the CA1 region of the dentate gyrus in a process mediated by glutamate, which activates the NMDA receptor that, in turn, induces extracellular signal regulated protein kinase (ERK) activation, followed by cAMP-responsive element-binding protein (CREB) phosphorylation, which persists for hours after LTP induction [176,177] (Figure 2). CREB promotes memory formation through the upregulation of neuronal excitability, memory trace cells activity, and successive events associated to memory storage and consolidation [178,179,180]. In addition, CREB phosphorylation induces the expression of BDNF, an important neurotrophin that plays a crucial role in synaptic activity transformation into long-term synaptic memories and general synaptic plasticity through interaction with tropomyosin related kinase (Trk) receptors, especially TrkB. BDNF/TrKB signaling activates downstream cascades, such as phosphatidylinositol 3-kinase (PI3-K)/Akt pathways, resulting in adequate cognitive process [178,179,180]. Thus, a blockade of NMDA receptors may result in cognitive deficits during ketamine bioavailability, as well as during withdrawal (Figure 2 and Figure 3). Indeed, as mentioned above, ketamine alters dopaminergic responses. There is evidence that, during abstinence, chronic ketamine consumers exhibited D1-receptor regional selective upregulation availability in dorsolateral prefrontal cortex, a phenomenon caused by chronic dopamine depletion in animal studies [181]. This fact indicates that the repeated use of ketamine impairs prefrontal dopaminergic signaling, which is a crucial step in working memory and executive function [181], as shown in Figure 2.

In turn, alcohol presents the NMDA receptor blockade as a primordial mechanism of action on the CNS; however, this toxicant can disrupt the homeostasis of several systems, and this can lead to cognitive impairment [96] (Figure 2). An extensive amount of works in the literature have shown the cognitive consequences elicited by ethanol consumption, affecting most processes related to cognition, even as a residual deleterious effect in long-term abstinence [182] (Figure 3).

Thus, it is reasonable to infer that the co-intoxication by ketamine plus ethanol may display profound consequences in cognitive domains and/or presents a risk augmented of mnemonic damage that is even higher than both substances isolated. Recently, it was demonstrated that ethanol aggravates ketamine-induced neurotoxicity through the downregulation of Akt, CREB, protein kinase A (PKA), and calmodulin-dependent kinase IV (CaMK-IV) on the cortex and hippocampus of adolescent rats under both substance effects [141] (Figure 2). These proteins are crucial for cell survival, and the repression of such signaling impairs neuronal viability and plasticity, thus affecting cognitive function [182,183,184]. Thus, a long-term negative impact on CREB and its downstream cascades can interfere directly with synapsis function and memory storage, even in withdrawal (Figure 3).

Finally, neuroadaptations resulting from ketamine-plus-ethanol withdrawal might be considered. We highlight the impact of events such as glutamatergic excitotoxicity and how subjacent apoptosis, neuroinflammation, oxidative stress, etc., might disrupt cognitive homeostasis (Figure 2). Findings related to ketamine and ethanol inducing similar damages support this theory [83,141,144,145,161,174,185]. It is noteworthy that these events have been extensively reported in the literature as components of pathophysiology of important cognitive disorders, such as Alzheimer’s disease [186,187,188] (Figure 2). Table 2 presents a compilation of studies related to the effects of ketamine or/and ethanol on the CNS in which polydrug consumption information was included.

## 4. Potential Pharmacological Interactions between Ketamine and Ethanol Use

The context of interaction between ethanol and ketamine is considerably complex. In fact, ethanol is the most consumed psychoactive drug worldwide, and it is culturally and legally accepted in most countries, with it mainly being linked to recreational contexts [96]. The broad variation in patterns of dose, frequency, and intermittence of consumption increases the complexity of analysis, since such patterns (i.e., acute effects vs. chronic consumption) induce opposite outcomes [6,7,8,9]. Ketamine, on the other hand, presents its use primarily linked to a medical context, as a sedative analgesic and general anesthetic, and more recently being proposed for affective disorders treatment [189,190]. However, ketamine has become popular as a recreational drug, used acutely or in a binge pattern; orally, inhaled, or injected; and alone or associated with alcoholic beverages or stimulants [191]. Each of these paradigms must be examined to identify potential synergistic points and risks related to this combination [11,12,13].

Pharmacokinetically, interactions between ethanol plus ketamine consumption are reasonable through competition for the CYP3A4 metabolic enzyme [192,193]. Although such an enzyme is not the primary route for alcohol metabolism, the consumption of a large amount of ethanol, as occurs in binge drinking pattern, elicits a metabolic enzymes saturation phenomenon, which may reduce ketamine metabolism, increasing ketamine bioavailability. On the other hand, ethanol chronic consumption induces CYP3A4 expression, increasing ketamine biotransformation and consequently reducing bioavailability. Therefore, investigations are necessary to elucidate the consequences of these interactions and their clinical relevance.

As highlighted in this review, systemic toxicity of both drugs is another point that deserves attention. Interestingly, both ethanol and ketamine present coincidental or synergistic deleterious effects on cardiorespiratory, hepatic, and urinary tracts [46,51,55,56,57,58,60,62,64,67,71,82,83]. Some of these damages are linked to concurrent pharmacological mechanisms on glutamatergic, noradrenergic, and cholinergic pathways’ modulation, resulting in biliary motility impairment, tachycardia, respiratory disability, and intraglomerular pressure. The ability to modulate ion channels, mitochondrial function, and oxidative metabolism interference is also common to both drugs, frequently triggering cell dysfunctions [194]. Ionic dysregulation is correlated with pulmonary edema, cardiac arrhythmias, and urinary dysfunction [195]. Mitochondrial dysfunction is associated with pro-oxidant effects that are related to inflammation, cell injury, apoptosis, and fibrosis in different systems [196,197]. Such harmful mechanisms shared by ethanol and ketamine use highlight the potential risk of this association that requires further investigation.

In CNS, the harmful potential effects of both of these drugs are mainly related to the concomitant ability to modulate glutamatergic and GABAergic pathways [198]. Different manifestations and symptoms may emerge, depending on the pattern of use (under effect of the drugs) or abstinence symptoms’ consequences. Considering that binge consumption consists of the main pattern among misusers, both ketamine and ethanol display a reduction of glutamatergic activity on NMDA receptors [198]. Additionally, ethanol potentiates GABAa receptors’ activity. Therefore, interference in formation of LTP processes, as well as reduced production of BDNF and Bcl-2, has been observed, impairing neuroplasticity and memory consolidation [199]. Accessory mechanisms such as the modulation of dopaminergic, serotonergic, cholinergic, and pathways, among other things, also may be shared by the drugs per se [19,65,96,108,158,159,160,181]. Considering that the isolated use of these substances, according to dose, induces distinct levels of cognition and perception disturbance, also identifying molecular synergistic potential, theoretically, the co-use may aggravate outcomes; this deserves further studies to be properly proved.

In an abstinence context, ketamine and ethanol also share deleterious mechanisms, involving overspread glutamatergic excitotoxicity, resulting in oxidative stress, mitochondrial dysfunction, neuroinflammation, and cell death induction [200,201]. These hazardous processes have been associated with genesis and poor outcomes of several CNS diseases, such as depression and anxiety [108,146,147,148,150]. Once again, the severity of manifestations might be influenced by dose, frequency, and intermittence of use, but the correlation between isolated use of ketamine or ethanol vs. anxiogenic or depressive-like behavior manifestation has already been demonstrated [25,202]. Based on all convergent targets divided by ethanol and ketamine use, which we have highlighted above in the present review, the probable risk of potentiation of CNS disorders in co-use circumstances is imminent and deserves further research.

## 5. Conclusions

The abuse of psychoactive substances has increased worldwide, catalyzed by economic and social factors, in addition to global calamities that affect humanity. In contrast to alcohol, which consists of the most consumed psychoactive drug, ketamine misuse has been augmented, attributed to its psychedelic activities. However, the co-ingestion of these drugs occurs among individuals who ignore the toxicological potentials of each drug and the risks of their combined use. Consequences of toxicological mechanisms shared by ketamine and ethanol might intensify several body systems’ damages, which can culminate in fatal outcomes. In the gastrointestinal system, concomitant abuse displays cirrhosis and hepatobiliary symptoms. Cardiorespiratory negative consequences, such as cardiotoxicity and respiratory depression, are the main evidence of toxicological hazardous effects. In the urinary system, renal dysfunction represents a critical outcome resulted from ketamine plus ethanol consume. Although the literature focused on the consequences of ketamine plus ethanol abuse is scarce, for the CNS, we can find extensive works in the literature. Similar mechanisms of action shared by ketamine and ethanol may potentiate the toxicological disturbance, resulting in schizotypy symptoms, depression, anxiety, and cognitive impairment. Additional studies are urgently needed to minimize the gap in the literature related to this co-abuse, as well as to explore future strategies to reduce the toxicological consequences that are caused by ketamine plus ethanol misuse.

As highlighted above, there are a variety of targets with potential for harmful interaction, compromising hepatobiliary, respiratory, cardiac, and urinary function, and thus placing users’ lives at risk. Similarly, several mechanisms related to changes in consciousness, cognition, and emotionality are shared by ketamine and ethanol, related to use and consequent repercussions in withdrawal. The consequences of such co-abuse deserves to be properly investigated in order to elucidate the toxicological mechanisms and clinical outcomes, clarify them to the community, and propose the development of new therapeutic strategies.

## Figures and Tables

**Table 1:** Effects reported in studies about ketamine and ethanol solely or combined on cardiovascular, respiratory, urinary, and hepatobiliary systems.

Drug(s)	Evaluation Condition	Study Information	Organ(s) or Body(s) System(s)	Main Effects Described	Possible Mechanisms	Reference
**Ketamine**	Under drug effects	Literature review	Cardiovascular and urinary	Acute: hypertension and tachycardia. Chronic: risk of hemorrhagic/ulcerative cystitis and obstructive nephropathy	Not investigated	[46]
**Ketamine**	Under drug effects	**Pattern of use:** chronic **Type:** clinical study **Finality of use:** recreational **Dose and use** **frequency:** not informed	Liver, biliary, urinary, and cardiorespiratory system	Under drug: cholestasis and biliary dilatation; hypotension; tachycardia and tachypnoea; bilateral hydronephrosis; acute renal failure; and increase in hepatic transaminases and alkaline phosphatase	Not investigated	[51]
**Ketamine**	Under drug effects	**Pattern of use:** acute **Type:** pre-clinical study **Finality of use:** not informed **Dose and use** **frequency:** 10, 50, 100, and 200 mM for 1, 6, or 24 h	Liver (in vitro)	Apoptosis and decreased cell viability	DNA fragmentation, mitochondrial membrane potential and adenosine triphosphate levels decrease; cytosolic cytochrome and caspase-9, -3, and -6 activities increase	[55]
**Ketamine**	Under drug effects	**Pattern of use:** acute **Type:** pre-clinical study **Finality of use:** not informed **Dose and use** **frequency:** a single administration of 180 mmol/L	Hepatobiliary	Strength of phasic gallbladder contraction decrease	NMDA blockade?	[56]
**Ketamine**	Withdrawal and drug effects	**Pattern of use:** chronic **Type:** clinical study **Finality of use:** recreational **Dose and use** **frequency:** not informed	Urinary system	Intractable dysuria, painful urination, and gross hematuria	Not investigated	[62]
**Ketamine**	Under drug effects	**Pattern of use:** chronic **Type:** clinical study **Finality of use:** recreational **Dose and use** **frequency:** frequency not informed; dose 100–200 mg (i.m.)/ intranasal route dose not informed	Cardiovascular system	Chest pain, palpitations, tachycardia, and hypertension	Not investigated	[60]
**Ketamine**	Under drug and withdrawal effects	Literature review	Cardiorespiratory system	Hypertension, tachycardia, and palpitations; respiratory toxicity, including depression and apnea	Authors propose that cardiovascular toxicity can result from reflex sympathetic activation	[64]
**Ketamine**	Under drug effects	**Pattern of use:** acute **Type:** pre-clinical study **Finality of use:** not informed **Dose and use** **frequency:** single dose of 100 mg (i.p.)	Cardiovascular system	Cardiotoxicity	Increase of microRNA-208a, accompanied by increased inflammation and oxidative stress, suppression of CHD9 and Notch1, and induction of p65 protein expression in vitro model	[66]
**Ketamine**	Not informed	**Pattern of use:** not specified **Type:** clinical study **Finality of use:** recreational **Dose and use** **frequency:** not informed	Hepatobiliary	Dilated common bile ducts and increase of alkaline phosphatase	Not investigated	[48]
**Ketamine**	Not informed	Literature review	Urinary system	Severe dysuria, painful hematuria, nocturia, pelvic pain, epithelial inflammation similar to chronic interstitial cystitis, and unilateral or bilateral hydronephrosis	Not investigated	[73]
**Ketamine**	Under drug effects	**Pattern of use:** chronic misuse **Type:** clinical study **Finality of use:** recreational **Dose and use** **frequency:** dose not informed, daily consume	Urinary system	Dysuria, painful hematuria, and post-voiding pain	Not investigated	[74]
**Ketamine**	Withdrawal effects	**Pattern of use:** chronic **Type:** pre-clinical study **Finality of use:** non-therapeutical **Dose and use** **frequency:** 100 or 300 mg/kg/day for 4 weeks	Urinary system	Bladder inflammation, interstitial nephritis, and increased lymphocytes in bladder submucosal layer	Voiding dysfunction by neurogenic damage and dysregulation of purinergic transmission	[75]
**Ketamine**	Under drug effects	**Pattern of use:** acute **Type:** pre-clinical study **Finality of use:** not declared **Dose and use** **frequency:** 1000 µM/1x	Cardiovascular system (in vitro)	Cerebral vasoconstriction	Blockade of Ca^2+^ activated K^+^ channels	[77]
**Ketamine**	Under drug effects	**Pattern of use:** acute **Type:** pre-clinical study **Finality of use:** not declared **Dose and use** **frequency:** 1, 10, 100, 1000, and 10,000 µM/1x	Cardiovascular system (in vitro)	Not specified	Inhibition of sarcolemmal adenosine triphosphate-sensitive potassium (K_ATP_) channels mediated by SUR subunit with specificity for cardiovascular K_ATP_ channels	[78]
**Ketamine**	Under drug effects	**Pattern of use:** chronic **Type:** pre-clinical study **Finality of use:** abuse **Dose and use** **frequency:** 100 mg/kg/day for 4, 8, and 16 weeks	Urinary system	Increased urination frequency and decreased bladder capacity after 8 weeks	Increased noncholinergic contractions and P2X1 receptor expression in bladder	[80]
**Ketamine**	Withdrawal effects	**Pattern of use:** chronic **Type:** pre-clinical study **Finality of use:** abuse **Dose and use** **frequency:** 30 mg/kg/day for 6 months	Urinary system	Relatively thinner bladder walls and infiltration of mononuclear cells, similar to clinical situation of interstitial cystitis	Decreased cholinergic neurons in urinary bladder by NMDA receptor overexpression	[81]
**Ketamine plus** **polydrugs**	Under drug and withdrawal effects	**Pattern of use:** chronic **Type:** clinical study **Finality of use:** recreational **Dose and use** **frequency:** not informed	Liver, biliary, and urinary system	Cystitis and urinary dysfunction; impaired liver function and biliary tree dilatation	Not investigated	[49]
**Ketamine plus** **polydrug**	Under drug effects	**Pattern of use:** chronic **Type:** clinical study **Finality of use:** recreational **Dose and use** **frequency:** not informed	Liver, biliary, cardiorespiratory, and urinary system	Common bile duct dilatation; increase in serum levels of hepatic transaminases; tachycardia; tachypnea and impaired respiratory function; overt hematuria associated with cramping abdominal pain, increased urinary frequency, dysuria, moderate bilateral hydronephrosis, and cystitis	Not investigated	[53]
**Ketamine plus other drugs**		**Pattern of use:** chronic **Type:** clinical study **Finality of use:** recreational **Dose and use** **frequency:** not informed	Liver and biliary system	Common bile duct dilatation, microscopic bile duct injury, and liver fibrosis	Not investigated	[54]
**Ketamine plus other drugs**	Under drug effects	**Pattern of use:** not informed **Type:** clinical study **Finality of use:** recreational **Dose and use** **frequency:** not informed	Cardiovascular and urinary system	Palpitations and chest pain, renal colic, urine leak, hematuria, and stranguria	Not investigated	[36]
**Ketamine plus other drugs**	Under drug and withdrawal effects	**Pattern of use:** chronic misuse **Type:** clinical study **Finality of use:** recreational **Dose and use** **frequency:** not informed	Respiratory system	Authors report the effect of ketamine in producing impairment of pharyngeal and laryngeal reflexes, diaphragm rigidity, and transient respiratory depression	Not investigated	[44]
**Ketamine plus other drugs**	Not specified	**Pattern of use:** chronic misuse **Type:** clinical study **Finality of use:** recreational **Dose and use** **frequency:** dose 0.125–5 g, frequency 3–7 days/week	Urinary system	Pain in lower abdomen; burning during urination and urination frequency increase; incontinence and presence of blood in urine	Not investigated	[38]
**Ketamine plus ethanol**	Under drug and withdrawal effects	**Pattern of use:** chronic **Type:** clinical study **Finality of use:** recreational **Dose and use** **frequency:** not informed	Liver and biliary system	Impaired liver function and possible damage to bile ducts	Not investigated	[50]
**Ketamine plus ethanol**	Withdrawal effects	Literature review	Liver, biliary, urinary, and cardiovascular system	Rats: dysuria, increased collagen fibers in hepatic parenchyma, and tachycardia Humans: signs and symptoms of cystitis, tachycardia, dysuria, and increase of liver fibrosis	Not investigated	[61]
**Ketamine plus ethanol**	Withdrawal and drug effects	**Pattern of use:** chronic **Type:** clinical study **Finality of use:** recreational **Dose and use** **frequency:** dose not informed; occasional alcohol intake and daily ketamine inhalation	Liver and biliary system	Increase in alkaline phosphatase and gamma-glutamyl transpeptidase; concentric periductal fibrosis around bile ducts of varying sizes, consistent with primary or secondary sclerosing cholangitis; interlobular bile ducts with thickened basement membranes, mild lymphocytic infiltrates, and mild ductular reaction	Not investigated	[63]
**Ethanol**	Under drug effects	Literature review	Liver	Liver fibrosis and cirrhosis, as a well as synergic interaction to infectious agents to induce liver injury	Production of hepatotoxins, activation of redox-sensitive transcription factors (i.e., nuclear factor kappa B (NF-κB), neutrophils, and other immune cells’ recruitment), circulating pro-inflammatory cytokines levels increase, and intestinal dysbiosis with hepatic repercussions	[57]
**Ethanol**	Under drug effects	Literature review	Liver	Liver fibrosis and cirrhosis, as a well as synergic interaction to infectious agents to induce liver injury	Production of hepatotoxins, induction of oxidative stress, activation of immune cells and circulating pro-inflammatory cytokines levels increase, deviation of lipid metabolism pathways, alteration of liver tissue remodeling factors activity, and intestinal dysbiosis with hepatic repercussions	[58]
**Ethanol**	Under drug effects	Literature review	Liver	Liver fibrosis	Acetaldehyde leads to generation of reactive oxygen species and activation of AP-1 and NF-kB transcription factors, resulting in pro-inflammatory cytokines’ release and increased survival and remodeling activity of stellate liver cells	[59]
**Ethanol**	Under drug effects	Literature review	Cardiovascular system	Alcoholic cardiomyopathy and holiday heart syndrome (chronic); arrhythmias (chronic and acute); and acute atrial fibrillation (acute)	Cardiovascular toxicity is a result of dysregulation in calcium and sodium conductance; beyond harmful effects on cardiac contractile function, in general after acute ethanol exposure	[67]
**Ethanol**	Under drug effects	Literature review	Respiratory system	Chronic consumption increases the risk of developing lung diseases, such as acute lung injury, pneumonia, and pulmonary fibrosis	Glutathione levels decrease and TGF-1 expression increase; changes in tissue remodeling and extracellular matrix in lung tissue; activation of matrix metalloproteinases (MMPs) in lungs, particularly MMP-2 and MMP-9; and increase in reactive oxygen species generation	[71]
**Ethanol**	Not specified	Literature review	Urinary system	Lower urinary tract symptoms	Urothelium vulnerability to alcohol and its metabolites, increased permeability, and lower urinary tract symptoms	[82]
**Ethanol**	Withdrawal effects	Meta-analysis of prospective chronic studies	Urinary system	Kidney damage is proportional to time of consumption and amount of alcohol, and there is a high prevalence of proteinuria in chronic users	Not investigated	[83]
**Ethanol**	Not informed	Systematic review and meta-analysis	Urinary system	Statistically significant inverse relationship of CKD in male adults with high alcohol consumption; and no significant association between high alcohol consumption and risk of developing proteinuria or end-stage renal disease	Not investigated	[84]
**Ethanol**	Withdrawal effects	**Pattern of use:** chronic **Type:** pre-clinical study **Finality of use:** abuse **Dose and use** **frequency:** ethanol 20% (*v*/*v*) daily for 24 weeks	Urinary system	Increase of blood pressure, uric acid, and albumin; and kidney changes	Activation of renin–angiotensin system (RAS), oxidative stress and increased sympathetic nerve activity	[85]
**Ethanol**	Under drug effects	**Pattern of use:** chronic **Type:** pre-clinical study **Finality of use:** abuse **Dose and use** **frequency:** ethanol 10% (*v*/*v*) daily for 12 weeks	Urinary system	Reduction in creatinine clearance and urea in urine; and urea serum increase	Not investigated	[86]
**Ethanol**	Under drug effects	**Pattern of use:** chronic **Type:** pre-clinical study **Finality of use:** abuse **Dose and use** **frequency:** ethanol 20% (*v*/*v*) daily for 12 weeks	Cardiovascular system	Blood pressure increase	Increased aortic inflammation; elevated angiotensin II levels; induction of NADPH oxidase, leading to endothelial injury; CuZn–SOD depletion; downregulation of endothelial NO-generating system; and impaired vascular vasodilatation in rats	[87]
**Ethanol**	Withdrawal effects	**Pattern of use:** chronic **Type:** pre-clinical study **Finality of use:** abuse **Dose and use** **frequency:** ethanol 20% (*v*/*v*) daily for 6 weeks	Urinary system	Increase in nitrite and nitrate levels and tubular necrosis in proximal tubules that is consistent in humans (acute tubular necrosis)	Reactive oxidative species generation and infiltration of polymorphonuclear cells	[88]
**Ethanol**	Under drug effects	**Pattern of use:** chronic **Type:** pre-clinical study **Finality of use:** abuse **Dose and use** **frequency:** ethanol 2 g/kg daily for 10 and 30 weeks	Urinary system	Kidney weight increase and new protein band with high molecular weight after 30 weeks of ethanol treatment.	Reduction of reduced glutathione/oxidized glutathione, kidney alcohol dehydrogenase activities increase, and distinct effects under antioxidant enzymes after 10 and 30 weeks	[89]
**Ethanol**	Under drug effects	**Pattern of use:** chronic **Type:** pre-clinical study **Finality of use:** abuse **Dose and use** **frequency:** ethanol 30% (*v*/*v*) daily for 2 months	Cardiovascular and urinary system	Heart size augment and reduction of glomerular filtration rate	Increase of catalase activity and decrease of lipid peroxidation in heart; opposite effects in kidney	[91]

**Table 2:** Effects reported in studies about ketamine and ethanol solely or combined on cognition, emotionality, and schizotypal parameters.

Drug(s)	Evaluation Condition	Study Information	CNS Function/ Disorder	Main Effects Described	Possible Mechanisms	Reference
**Ketamine**	Under drug and withdrawal effects	**Pattern of use:** acute **Type:** clinical study **Finality of use:** abuse **Dose and use** **frequency:** 2 mg/kg daily	Psychosis	Impaired on semantic memory tasks; higher levels of dissociation and schizotypal symptoms	Not investigated	[24]
**Ketamine**	Withdrawal effects	**Pattern of use:** chronic misuse **Type:** clinical study **Finality of use:** abuse **Dose and use** **frequency:** dose not informed; frequency— more than 4 times a week.	Cognition and depression	Short- and long-term memory impairments, vulnerability to dangers behavior, and depressive symptoms	Not investigated	[40]
**Ketamine**	Withdrawal effects	**Pattern of use:** chronic **Type:** clinical study **Finality of use:** abuse **Dose and use** **frequency:** dose not informed; frequent use	Cognition and depression.	Frequent use: reduced psychological well-being and broad range of cognitive impairments. Infrequent use: increased symptoms of thought disorder, delusions, and dissociation, but not cognitive impairment	Not investigated	[39]
**Ketamine**	Under drug effects	**Pattern of use:** acute **Type:** clinical study **Finality of use:** not specified **Dose and use** **frequency:** single administration of 0.4 mg/kg and 0.8 mg/kg.	Psychosis	Increased schizophrenic and dissociative symptoms after ketamine use; psychotomimetic effects of ketamine are detectable on clinical scales	Not investigated	[103]
**Ketamine**	Under drug effects	**Pattern of use:** acute **Type:** clinical study **Finality of use:** clinical **Dose and use** **frequency:** 0.5 mg/kg infused over 40 min	Emotionality	Subacute increase of prefrontal connectivity associated to antidepressant response	Ketamine seems activate prefrontal glutamate neurotransmission, contributing to transient psychotomimetic effects and delayed and sustained antidepressant effects	[114]
**Ketamine**	Under drug effects	**Pattern of use:** acute **Type:** clinical study **Finality of use:** subanesthetic **Dose and use** **frequency:** 0.3 mg/kg for 2 weeks	Psychosis	Increase in schizotypal symptoms	Flow activation on anterior cingulate and prefrontal cortex; decreased flow activation on visual cortex and hippocampus; and abnormal glutamatergic transmission involved on pathophysiology of psychotic symptoms	[117]
**Ketamine**	Not specified	Literature review	Cognition and psychosis	Cognitive impairment; psychotic and negative symptoms	Reduction of NMDA receptors related to negative symptoms	[108]
**Ketamine**	Under drug effects	**Pattern of use:** acute **Type:** clinical study **Finality of use:** non-therapeutical **Dose and use** **frequency:** 15 mg/kg for 5 min to induce psychopathological effects, followed by dose of 0.014 mg/kg/min for 90 min	Emotionality and psychosis	Affect changes, illusions, hallucinations, and ego dissolution	Inhibition of NMDA receptors to increase in DA levels in striatum, inducing euphoria- and mania-related features	[119]
**Ketamine**	Under drug and withdrawal effects	**Pattern of use:** acute **Type:** pre-clinical study **Finality of use:** not informed **Dose and use** **frequency:** 2, 10, and 20 mg/kg (i.p(i.p.), or once, or twice; or 25 mg/kg for 7 days	Cognition	Highest ketamine dose elevates levels of errors of omission in attentional tasks, whereas disrupted attentional performance during pre-treatment period	Increase in cortical acetylcholine release	[121]
**Ketamine**	Under drug effects	**Pattern of use:** acute **Type:** pre-clinical study **Finality of use:** subanesthetic **Dose and use** **frequency:** single dose 150 mg/kg	Schizotypal behaviors	Suppression of high-frequency EEG activity and disruption of cortical coherence	Increased concentrations of cortical ACh in active arousal systems in setting of unconscious state	[122]
**Ketamine**	Under drug effects	**Pattern of use:** not informed **Type:** pre-clinical study **Finality of use:** not informed **Dose and use** **frequency:** a single administration of 0.1 nM to 10,000 nM	Psychosis (in vitro)	Biological events related to schizophrenia	Ketamine binds to D2 receptors, increasing dopamine neuro-availability; and antagonism of NMDA receptors induces schizotypy	[123]
**Ketamine**	Under drug effects	**Pattern of use:** acute **Type:** pre-clinical study **Finality of use:** not specified **Dose and use** **frequency:** a single administration of 0.01 a 100 µM	Psychosis (in vitro)	Partial agonism on D2, and 5-HT_2_ (on a smaller scale) receptors	Ketamine binds to D2 receptors, increasing the neuro-availability of dopamine; small affinity to 5-HT_2_ receptors; and other mechanisms induce non-selective multi-system neurochemical perturbation	[124]
**Ketamine**	Under drug effects	**Pattern of use:** acute **Type:** pre-clinical study **Finality of use:** therapeutical **Dose and use** **frequency:** 30 mg/kg and local administration of 0.1 mM	Emotionality	Increased serotonin release in medial prefrontal cortex	Increase of serotonin release on medial prefrontal cortex through cholinergic neurons projected pedunculopontine tegmental nucleus to dorsal raphe nucleus	[125]
**Ketamine**	Not specified	Literature review	Psychosis	Schizotypal symptoms	Glutamatergic hypoactivity through antagonism of NMDA receptors may be associated with schizophrenia manifestation	[126]
**Ketamine**	Under drug effects	Literature review	Cognition	Abusive use of ketamine induces cognitive damage	NMDA receptors blockade on gamma-aminobutyric acid (GABA) neurons on thalamic reticular nucleus leads to disinhibition of dopaminergic neurons and increased dopamine release	[142]
**Ketamine**	Withdrawal effects	**Pattern of use:** acute **Type:** pre-clinical study **Finality of use:** abuse **Dose and use** **frequency:** 10 mg/kg for 3 days	Cognition and emotionality	Memory impairment, anxiogenic and depressive behavior	Oxidative stress in hippocampus	[25]
**Ketamine**	Withdrawal effects	**Pattern of use:** chronic **Type:** clinical study **Finality of use:** abuse **Dose and use** **frequency:** 3.4 g/day; frequency varied between 1 time per day, more than 4 times per week, and less than 4 times per week	Cognition and emotionality	Depressive, anxiogenic, and psychotic symptoms	Not investigated	[165]
**Ketamine**	Under drug and withdrawal effects	**Pattern of use:** acute **Type:** pre-clinical study **Finality of use:** anesthetic and subanesthetic **Dose and use** **frequency:** 2 mg/kg daily for 2 days	Anxiety	Doses employed have no effects on anxiety- or panic-related behaviors	Not investigated	[167]
**Ketamine**	Withdrawal effects	**Pattern of use:** chronic **Type:** clinical study **Finality of use:** abuse **Dose and use** **frequency:** not informed	Dissociative symptoms	Greater affective symptoms and perceptual disturbances	Not investigated	[93]
**Ketamine**	Withdrawal effects	**Pattern of use:** chronic **Type:** clinical study **Finality of use:** abuse **Dose and use** **frequency:** not informed	Cognition, emotionality and schizotypal symptoms	Semantic memory deficiencies decrease but are reversible with marked reduction in use; impairment in episodic memory and attentional functioning; schizotypal symptoms and perceptual distortions may persist after discontinuing ketamine use	Not investigated	[104]
**Ketamine**	Withdrawal effects	**Pattern of use:** acute **Type:** clinical study (case report) **Finality of use:** anesthetic **Dose and use** frequency 650 mg/kg to 15 mg/kg for 7 days	General behavioral alterations	Fever, sleep inversion, restlessness, and drooling (24 and 48 h after withdrawal); 17 days later, behavior and cognitive impairment, including aggression and language deficits, in addition to affecting motor skills	Not investigated	[175]
**Ketamine**	Withdrawal effects	**Pattern of use:** acute **Type:** pre-clinical study **Finality of use:** anesthetic **Dose and use** **frequency:** single dose of 20 mg/kg subcutaneously	Cognition	Impairment in short-term recognition memory	Decreased cellular viability in hippocampus, and long-term increase in hippocampal glutamate uptake. Prevention of vulnerability to glutamate-induced neurotoxicity in frontal cortex of adult rats	[174]
**Ketamine**	Under drug effects	**Pattern of use:** chronic **Type:** clinical study **Finality of use:** abuse **Dose and use** **frequency:** at least an average use of one vial per week or more over the last 3 months	Memory	Working memory not affected	Dorsolateral prefrontal cortex D1 receptor upregulation; binding potential upregulation significantly correlated to number of vials of ketamine used per week.	[181]
**Ethanol**	Withdrawal effects	**Pattern of use:** chronic **Type:** clinical study **Finality of use:** abuse **Dose and use** **frequency:** not informed	Psychosis	Hallucinations of schizophrenic origin and hallucinations from alcohol are very similar	Not investigated	[132]
**Ethanol**	Withdrawal effects	**Pattern of use:** chronic **Type:** clinical study **Finality of use:** abuse **Dose and use** **frequency:** 200 g/day for 30 years	General behavioral alterations	Finger tremor, mildly altered liver enzymes, and decreased regional cerebral blood flow	Not investigated	[136]
**Ethanol**	Withdrawal effects	**Pattern of use:** chronic **Type:** clinical study **Finality of use:** abuse **Dose and use** **frequency:** 400 g/day of alcohol for a long time (undefined)	Psychotic state	Hallucinations	Damage to thalamic structures associated with manifestation of hallucinations	[137]
**Ethanol**	Withdrawal effects	**Pattern of use:** chronic **Type:** clinical study **Finality of use:** abuse **Dose and use** **frequency:** unreported dose and consume frequency at least 10 years	Psychotic state	Verbal hallucinations, hallucinatory delusions, and affective frustration (mainly alarm and fear) in a state of clear consciousness	Acute alcoholic hallucinosis is linked to changes in excitatory and inhibitory transmission in the brain	[138]
**Ethanol**	Withdrawal effects	**Pattern of use:** acute **Type:** clinical study **Finality of use:** abuse **Dose and use** **frequency:** unreported dose; ex-user patients undergoing treatment	Psychotic state	Chronic alcohol consumption induces hallucinogenic events, and it alters plasmatic concentration of tyrosine, tryptophan, and phenylalanine	Amino acid imbalances result in decreased brain serotonin levels and increased brain dopamine, inducing hallucinatory experiences	[139]
**Ethanol**	Under drug or withdrawal effects	**Pattern of use:** chronic **Type:** pre-clinical study **Finality of use:** abuse **Dose and use** **frequency:** 2 g/kg/day or 10%; daily frequency	Rewarding system	Broader effects on dopaminergic system in voluntary ethanol intake model	Increased dopamine degradation, influencing the reward system	[140]
**Ethanol**	Under drug and withdrawal effects	Literature review	General behavioral alterations	Withdrawal symptoms, delirium tremens, Wernicke–Korsakoff syndrome, and fetal alcohol syndrome	Acute effects of ethanol disrupt glutamatergic neurotransmission by NMDA blockade. Prolonged inhibition of NMDA receptor results in development of supersensitivity. Acute absence of ethanol increases postsynaptic neurons activity (i.e., noradrenergic system and glutamate-induced excitotoxicity)	[143]
**Ethanol**	Under drug effects	Literature review	Reward system	Binge drinking induces decreased reward neurocircuitry function and recruitment of anti-reward/stress mechanisms	Ethanol interacts with NMDA, GABA_A_, glycine, 5-HT, and nicotinic receptors, as well as L-type Ca^2+^ channels and G protein-activated internal rectifier K^+^ channels of neurotransmitters/neuropeptides leading to typical acute behavioral effects of alcohol	[144]
**Ethanol**	Withdrawal effects	Literature review	General behavioral alterations	Anxiety, depression, tremors, rigidity, hyperactivity, convulsion, coma, and even death	Intense generation of reactive oxygen species and activation of stress-responding protein kinases	[145]
**Ethanol**	Under drug and withdrawal effects	Literature review	CNS adaptations	Brain excitotoxicity	Hyperexcitability after alcohol withdrawal may contribute to excitotoxicity. Alterations in function and/or expression of glutamate, GABA, and voltage-activated calcium channels contribute to hyperexcitability	[153]
**Ethanol**	Under drug and withdrawal effects	Literature review	General behavioral alterations	Depressive episodes, severe anxiety, insomnia, suicide, and abuse of other drugs	Not investigated	[154]
**Ethanol**	Under drug and withdrawal effects	Literature review	CNS impairments	Acute: excitement, ataxia, and lethargy. Chronic: cognitive, emotional, and motor disturbances. Withdrawal: autonomic hyperactivity, tremor, anxiety, restlessness, seizures, hallucinations, and delirium	Ethanol stimulates microglia, inducing neuroinflammation that triggers neuropathogenic processes	[155]
**Ethanol**	Withdrawal effects	**Pattern of use:** chronic **Type:** clinical study **Finality of use:** abuse **Dose and use** **frequency:** unreported dose and frequency	Cognition and emotionality	Depressive disorders, anxiety, sleep disturbance, impairment of cognitive ability, and increased plasma macrophage-derived chemokine	Macrophage-derived chemokine is associated with alcoholism, phobia, and interpersonal sensitivity	[156]
**Ethanol**	Withdrawal effects	**Pattern of use:** chronic **Type:** pre-clinical study **Finality of use:** abuse **Dose and use** **frequency:** 4% v.o. for 26 months	Anxiety	Social isolation and increased cortisol	Not investigated	[171]
**Ethanol**	Withdrawal effects	**Pattern of use:** acute **Type:** pre-clinical study **Finality of use:** abuse **Dose and use** **frequency:** vapor exposure for 7 days; blood alcohol levels 127 mg%	Endocrine regulation	Not specified	Hypothalamic–pituitary adrenal axis activity alterations	[172]
**Ethanol**	Under drug effects	**Pattern of use:** acute **Type:** clinical study **Finality of use:** abuse **Dose and use** **frequency:** a single dose of 0.8 g/kg	Cognition and emotionality	Negative effects on several dimensions of mood; impaired verbal memory; and dysphoria	Norepinephrine mediates behavioral alterations ethanol-induced (i.e., dysphoria), while serotonin provokes opposite activity	[158]
**Ethanol**	Under drug effects	**Pattern of use:** acute **Type:** pre-clinical study **Finality of use:** abuse **Dose and use** **frequency:** a single administration of 3.5 g/kg (i.p.)	CNS monoamine dependent functions	Not investigated	Mesolimbic and nigrostriatal dopaminergic systems’ hyperactivation, increasing DA release and catabolism	[159]
**Ethanol**	Under drug effects	**Pattern of use:** acute **Type:** pre-clinical study **Finality of use:** abuse **Dose and use** **frequency:** 1, 2, 3, and 4 g/kg for 7 days	CNS monoamine dependent functions	Not investigated	Dopamine, NE, and metabolite’s levels decrease on dorsal raphe and *Locus coeruleus*	[160]
**Ethanol**	Not informed	Literature review	General behavioral alterations	Psychomotor depression, difficulties in information storage and logical reasoning, motor incoordination, stimulation of reward system	Direct action on GABA, glutamate, and endocannabinoids systems; indirect action on limbic and opioid system; action on calcium channels, potent and proteins regulated by GABA hippocampus, in addition to central actions not mediated by vitamin B1 deficiency	[161]
**Ethanol**	Under drug and withdrawal effects	Literature review	Cognition	Nutritional disorders and dementia	Loss of hippocampal CA1 and CA3 pyramidal neurons, mossy fiber-CA3 synapses and dentate granule cells, and cholinergic neurons in basal forebrain; pathological neuroadaptations (i.e., excitatory/inhibitory neurotransmitters balance and oxidative stress)	[182]
**Ketamine plus ethanol**	Under drug effects	**Pattern of use:** sub-chronic **Type:** pre-clinical study **Finality of use:** abuse **Dose and use** **frequency:** 2 or 4 g/kg orally of ethanol plus 30 mg/kg i.p. ketamine for 14 days	Schizotypy	Increased activity, stereotyped behavior, ataxia, and morphological changes, as well as severe neurotoxicity	Altered behaviors were associated with alcohol-induced increases in ketamine-induced higher levels of Glu and DA in cortex and hippocampus	[141]
**Ketamine plus ethanol**	Under drug effects	**Pattern of use:** acute **Type:** pre-clinical study **Finality of use:** abuse **Dose and use** **frequency:** 10% ethanol solutions plus 0.28 mg/mL ketamine for 35 days, for 1 h	Anxiety	Ethanol: anxiogenic-like behavior. Ketamine: anxiolytic-like behavior. Ketamine + ethanol: absence of effect	Ketamine interfered in development of tolerance to anxiolytic effects of ethanol through modulation of several subsystems targeted by both drugs	[173]
**Ethanol plus other drugs**		**Pattern of use:** chronic **Type:** pre-clinical study **Finality of use:** abuse **Dose and use** **frequency:** 3 g/kg/day, 3 times a week for 5 weeks	Cognition and emotionality	Cognitive impairment; depressive and anxiety-like behavior	Alteration in peripheral markers of oxidative stress: decrease in nitrite levels; increase of sulfhydryl groups, MDA levels, superoxide dismutase, and catalase activity	[157]

## Data Availability

Not applicable.
